# To effectively reduce violence against women living with HIV, we must make healthcare systems places of safety

**DOI:** 10.1002/jia2.25851

**Published:** 2021-11-24

**Authors:** Sophie O. Brion, Jody L. Fredericks, Charity T. Mkona, Sita Shahi

**Affiliations:** ^1^ International Community of Women Living with HIV Global Kampala‐Hoima Uganda; ^2^ International Community of Women Living with HIV Global Cape Town South Africa; ^3^ International Community of Women Living with HIV South Africa Lilongwe Malawi; ^4^ International Community of Women Living with HIV ‐ Asia Pacific Kathmandu Nepal

As the COVID‐19 pandemic continues to evolve, a “shadow pandemic” of intensified intimate partner violence and sexual assault has unfolded for women worldwide [1]. Although data on the scale of the violence are still emerging, concerning early reports include a 24% increase in reported rapes in Uganda [2], 30–45% increases in domestic violence cases in France and Brazil, respectively, and a 130% increase in calls to domestic violence hotlines in Colombia [3]. Women living with HIV and women from key populations have experienced unique and increased vulnerabilities to intimate partner violence due to pandemic and lockdown‐related challenges in maintaining confidentiality, accessing and adhering to treatment, care and support, particularly sexual and reproductive health and maternal care. Women living with HIV have also reported increased instances of forced disclosure of HIV status, and increased stigma and discrimination [4, 5, 6]. The rise in violence and the inadequacy of available support and services have catalyzed public discourse about strengthening responses to intimate partner violence, with health systems envisioned as essential sources of help and support for women experiencing gender‐based violence [7]. However, these systems have not reliably been places of safety for women living with HIV.

Even before the pandemic, women living with HIV experienced disproportionate levels of violence in healthcare settings related to their HIV status, namely, denials of care, stigma and discrimination, and obstetric violence [8]. Obstetric violence is a specific form of gender‐based violence experienced by women during pregnancy, childbirth and the postpartum period in healthcare settings, including disrespectful and abusive treatment, physical abuse and violations of bodily integrity and autonomy, such as forced or coerced medically unjustified sterilization, abortion and Caesarian section [9,10]. The World Health Organization has identified women living with HIV as particularly likely to experience this type of disrespectful and abusive treatment during maternal healthcare [11, 12]. Networks of women living with HIV have reported hostile attitudes, lack of informed consent, and forced and/or coercive sterilization in nearly 40 countries [13].

Obstetric violence documented during the pandemic includes denials of maternal healthcare, lack of access to contraception and safe abortion, neglect, abandonment, increases in stigma and discrimination, and procedures performed without informed consent, including efforts to speed up and induce labour, Caesarian section and episiotomies [14, 15, 16]. Less well‐quantified are the increased adverse impacts on women living with HIV of COVID‐19 pandemic‐related deprioritization of sexual and reproductive health services [4–6, 8]. Experiences of abuse and discrimination in healthcare settings create barriers to care for women living with HIV who may avoid seeking services. Nevertheless, the healthcare system remains central to envisioned responses to gender‐based violence, even while making insufficient progress in addressing violence perpetrated within its services.

To effectively respond to intimate partner violence and to realize the potential of healthcare systems as places of safety for women living with HIV, we can no longer afford to give short shrift to violence in healthcare settings. We must take decisive action to shift the status quo. We recommend the following three steps:

First, we must transform harmful power dynamics in healthcare settings. Like intimate partner violence, stigma, discrimination and obstetric violence experienced by women living with HIV in healthcare settings are manifestations of pervasive gender inequality and harmful and gendered power dynamics, which undermine women's autonomy, bodily integrity and human rights. For healthcare settings to truly serve as places of safety for women living with HIV, we must take a women‐centred approach to reform harmful attitudes and gender norms. Transforming these power dynamics requires putting women's agency and self‐determination at the centre of health services. Part of the equation is urgent scale‐up and investment in training of healthcare providers, in stigma reduction efforts, and in the training and sensitization of women living with HIV about their rights.

Second, we must increase accountability and take reports of human rights violations seriously. Stakeholders must take women's rights violations seriously and respond adequately with a process that seeks reform, accountability, justice and remedy for women experiencing these violations. We have seen positive progress in South Africa and Chile. In South Africa, the Commission for Gender Equality (CGE) released an investigation report, in 2019, into a complaint brought by the International Community of Women Living with HIV (ICW), ICW Southern Africa and allies on behalf of more than 48 women living with HIV [17]. The report confirmed the widespread incidence of coercion of women living with HIV into sterilization due to their HIV status in state hospitals. The CGE recommended that the South African National Department of Health provide redress to the victims, engage in policy reform and hold actors accountable. ICW engaged in 7 years of both advocacy and legal representation to reach this result. Similarly, in Chile, the government entered a “friendly settlement” in 2021 with Francisca, a woman forced into sterilization due to her HIV status [18]. The settlement includes policy reforms to protect against forced sterilization and discrimination. The Centre for Reproductive Rights and Vivo Positivo represented Francisca for over a decade to achieve this outcome. Proper investigation of human rights violations is a critical first step in confronting gender‐based violence in institutional settings [19].

Third, we must invest in networks of women living with HIV and key populations. During the pandemic, networks of women living with HIV and key populations responded to critical gaps in services, delivering treatment, food, supplies, accurate information, psychosocial support and raising the alarm on challenges and unmet needs of community members. These networks must be recognized for their essential support role, precise positioning and insights into the healthcare system. Despite this vital function, they are chronically and perpetually underfunded. Investments in networks are long‐overdue and must support community‐led monitoring and evaluation of services, including prevention of vertical transmission services [3] and long‐term support for networks to document and confront rights violations, address stigma, discrimination and abuse in healthcare settings, and to hold healthcare providers accountable [19].

The COVID‐19 pandemic has presented an urgent call to action to recognize, confront and transform the entrenched gender inequality, misogyny and harmful power imbalances that intensify the vulnerabilities of women living with HIV to violence, whether interpersonal or institutional. Will we answer the call?

## COMPETING INTERESTS

The authors declare no competing interests.

## AUTHORS’ CONTRIBUTIONS

SB and JLF contributed to primary research and writing. SB and JLF provided legal advocacy and technical support for the ICW‐led complaint to the South African Commission on Gender Equality. CS and SS contributed to supplementary research and provided key conceptual elements. All authors have read, reviewed, contributed to and approved the final manuscript for this Viewpoint.

## FUNDING

No funding was provided for this article.

## DISCLAIMER

The viewpoints expressed in this article are solely that of the authors.

## Data Availability

Data sharing is not applicable to this article as no new data were created or analyzed in this study.
